# Proteome Changes Driven by Phosphorus Deficiency and Recovery in the Brown Tide-Forming Alga *Aureococcus anophagefferens*

**DOI:** 10.1371/journal.pone.0028949

**Published:** 2011-12-14

**Authors:** Louie L. Wurch, Erin M. Bertrand, Mak A. Saito, Benjamin A. S. Van Mooy, Sonya T. Dyhrman

**Affiliations:** 1 Biology Department, Woods Hole Oceanographic Institution, Woods Hole, Massachusetts, United States of America; 2 Department of Marine Chemistry & Geochemistry, Woods Hole Oceanographic Institution, Woods Hole, Massachusetts, United States of America; US Dept. of Agriculture – Agricultural Research Service (USDA-ARS), United States of America

## Abstract

Shotgun mass spectrometry was used to detect proteins in the harmful alga, *Aureococcus anophagefferens*, and monitor their relative abundance across nutrient replete (control), phosphate-deficient (−P) and −P refed with phosphate (P-refed) conditions. Spectral counting techniques identified differentially abundant proteins and demonstrated that under phosphate deficiency, *A. anophagefferens* increases proteins involved in both inorganic and organic phosphorus (P) scavenging, including a phosphate transporter, 5′-nucleotidase, and alkaline phosphatase. Additionally, an increase in abundance of a sulfolipid biosynthesis protein was detected in −P and P-refed conditions. Analysis of the polar membrane lipids showed that cellular concentrations of the sulfolipid sulphoquinovosyldiacylglycerol (SQDG) were nearly two-fold greater in the −P condition versus the control condition, while cellular phospholipids were approximately 8-fold less. Transcript and protein abundances were more tightly coupled for gene products involved in P metabolism compared to those involved in a range of other metabolic functions. Comparison of protein abundances between the −P and P-refed conditions identified differences in the timing of protein degradation and turnover. This suggests that culture studies examining nutrient starvation responses will be valuable in interpreting protein abundance patterns for cellular nutritional status and history in metaproteomic datasets.

## Introduction

*Aureococcus anophagefferens* is the phytoplankton species responsible for harmful brown tides that have caused extensive damage to a number of coastal ecosystems in the Eastern United States [1]. Brown tides have led to a collapse of the Long Island scallop industry and caused substantial losses to eelgrass habitats [2], [3], [4]. Furthermore, brown tides are becoming more frequent and widespread, as evidenced by brown tides now occurring in South Africa [1]. Due to its negative impacts and the regular and widespread occurrence of blooms, *A. anophagefferens* has become a broadly studied harmful algal bloom (HAB) species (see reviews [1], [5] and is the first HAB species to have its genome sequenced [6].

Past studies have suggested that brown tides are driven by periods of low dissolved inorganic nitrogen (DIN) and low dissolved inorganic phosphorus (DIP) availability [7]–[12]. Although studies of phosphorus (P) effects on bloom dynamics are more limited than those of nitrogen (N), field observations from brown tides have shown significant reductions in dissolved organic phosphorus (DOP) concentrations during peak *A. anophagefferens* cell densities [11]. Analysis of the genome suggests that *A. anophagefferens* has the capacity to utilize P from a variety of organic sources, including esters, diesters, and nucleotides [6]. In culture, *A. anophagefferens* can utilize nucleotide DOP such as adenosine monophosphate (AMP) as a sole P source, which is consistent with genome observations [6], [13]. When DIP becomes deficient, *A. anophagefferens* exhibits a broad transcriptional response, up-regulating a variety of these P-scavenging genes such as a phosphate transporter, 5′-nucleotidase, and alkaline phosphatase, where the latter two are important enzymes used by phytoplankton to access P from the DOP pool [13]. These data, combined with field observations, suggest that DOP could be important in controlling bloom persistence and decline.

Genome and transcriptome sequencing efforts have provided key insights into the metabolic potential of harmful phytoplankton species [14], [15]. Despite the value of these sequencing efforts, studies in humans have demonstrated that much of the transcribed genome is never translated [16], suggesting that transcriptome analyses may overestimate actual cellular processes and physiological responses to changes in nutrient availability. Mass spectrometry-based proteomics allow direct measurements of changes in an organism's protein pool, thus more accurately assessing the arsenal of chemical responses these organisms employ for growth under different physiological conditions. Proteomics is also a valuable compliment to nucleic acid sequencing approaches as a tool for examining how molecular-level pathways drive physiological responses. Recently, mass spectrometry-based proteomic approaches have successfully been employed to analyze primary metabolic and biosynthetic pathways in the diatom *Thalassiosira pseudonana* [17] and the picoeukaryote *Ostreococcus tauri* [18] as well as the diazotrophic unicellular marine cyanobacteria *Crocosphaera watsonii* [19]. Similar proteomic techniques are currently being applied to *in situ* ocean communities and have allowed for the direct observation of expressed proteins from mixed microbial consortia [20], [21]. These metaproteomic approaches revealed that transporters dominate the pool of identifiable proteins in low nutrient environments on ocean-wide scales [20], [21]. However, without detailed information on protein regulation, it is difficult to link the abundance of particular proteins, like these transporters, to cellular physiology or a cell's geochemical environment.

Herein, shotgun mass spectrometry was used to identify protein abundances in *A. anophagefferens* in nutrient replete (control) and phosphate-deficient (−P) treatments. In order to examine the timing of these responses, proteins were also assayed in a phosphate-refed (P-refed) treatment, where replete levels of phosphate were added to −P cells over a 24-hour period. Protein abundances were compared between two treatments using spectral counting and compared to transcript expression patterns from a previous study [13].

## Results and Discussion

Shotgun mass spectrometry was used to identify proteome responses to P deficiency. A total of 3,431 unique peptide identifications (Table S1) were made from 214,913 total spectra, with a false discovery rate of 0.6%. From these data, 641 unique proteins (Table S2) were detected (see methods for description of statistical analyses). Although most of these proteins could be assigned a putative function, 37 could not and are listed as either hypothetical or predicted proteins (Table S2). A large percentage of the 641 proteins were annotated as ribosomal (13.3% or 85 proteins, Table S2).

There were 46 different light harvesting complex (LHC) proteins detected out of the 62 encoded in the genome [6] (Table S2). This is far more than detected in the proteome of the diatom *T. pseudonana* under optimal growth conditions, where a total of 14 different LHC homologues were identified [17]. *A. anophagefferens* is well adapted to low light conditions, reaching maximum growth rates at lower irradiances than its algal competitors, including the diatoms *T. pseudonana*, *Phaeodactylum tricornutum* and picoeukaryotes *Ostreococcus tauri* and *O. lucimarinus* [6]. This is consistent with the observation that *A. anophagefferens* has more unique LHC genes encoded in its genome than its algal competitors [6], and that these genes are translated.

### Differential protein abundance

**Figure 1:**
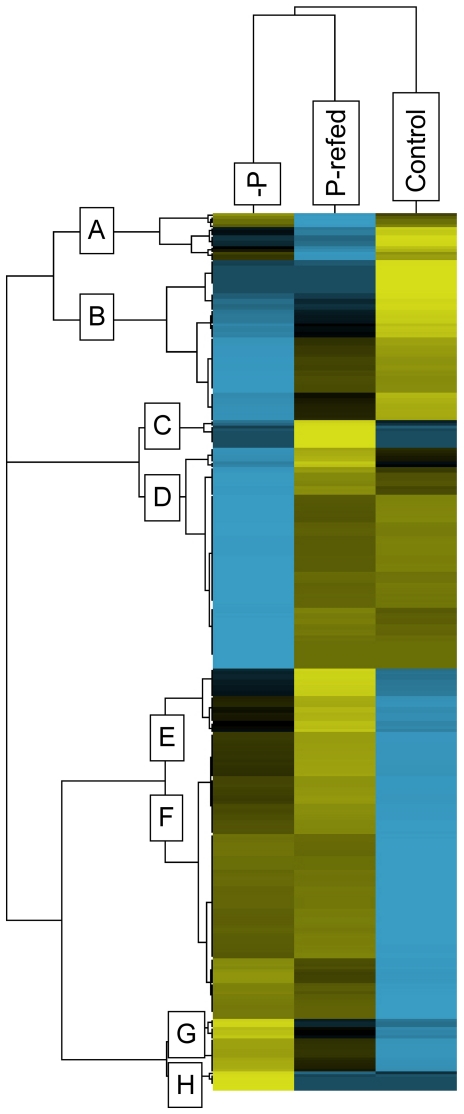
Patterns of all differentially abundant proteins. Hierarchical cluster analysis of the 318 proteins classified as differentially abundant. This analysis groups proteins by similarity of patterns. The spectral counts for each protein were averaged across treatments (−P, P-refed, control). Yellow indicates higher abundance than the mean while blue indicates reduced abundance relative to the mean. Black indicates no difference from the mean. The intensity of the color is indicative of the degree of difference from the mean, with brighter colors displaying stronger differences. Letters indicate clusters of similar pattern.

The abundance of the 641 proteins detected in this study were compared among treatments using spectral counting techniques. Out of the 641 proteins detected in this study, 49.6% (318 proteins) were differentially abundant in at least one treatment (control, −P, P-refed) relative to the other two based upon abundance score (see methods for description of statistical tests used to determine differentially abundant proteins and supplementary tables for individual *p*-values for all proteins) (Figure S1, Table S3). These 318 proteins were hierarchically clustered in order to group proteins with similar abundance patterns (Figure 1). The −P and P-refed treatments clustered together meaning the proteome of the P-refed treatment more closely resembled the proteome from the −P treatment than it did the control. Therefore, starting from a P-deficient state, 24 hours was not enough time for *A. anophagefferens* to return to a replete nutrient proteome composition. The proteins grouped together into eight distinct regulation patterns across the three treatments (A–H; Figure 1).

### Highest abundance in control

There were 75 proteins that were more abundant in the control condition relative to the −P and P-refed treatments (cluster A–B, Figure 1) and so are repressed during P deficiency. It appears that once phosphate is added to the −P cells, either these proteins remain repressed or there is a delay in their synthesis. The most abundant protein detected in this study, the large subunit of ribulose-1,5-bisphosphate carboxylase/oxygenase (RuBisCO), fell into this category (NCBI #: 242620086) and was about 3.6-fold less abundant under −P conditions (Table S3). Also abundant in this study was a small chain RuBisCO protein (NCBI #: 242620087). This protein was down-regulated 4.2 fold under −P (Table S3). In the diatom *T. pseudonana*, a RuBisCO large subunit was among the most abundant proteins detected under optimal growth conditions as well [17]. Although carbon fixation was not specifically examined in this study, these protein abundance results suggest that carbon fixation is likely reduced when P is deficient in *A. anophagefferens*. In the P-refed treatment, both the RuBisCO large and small subunit proteins were more abundant than the −P treatment, but still low relative to the control. Thus carbon fixation likely increases after P deficiency is alleviated, but 24 hours was not enough time for carbon fixation in cells to fully recover.

**Figure 2:**
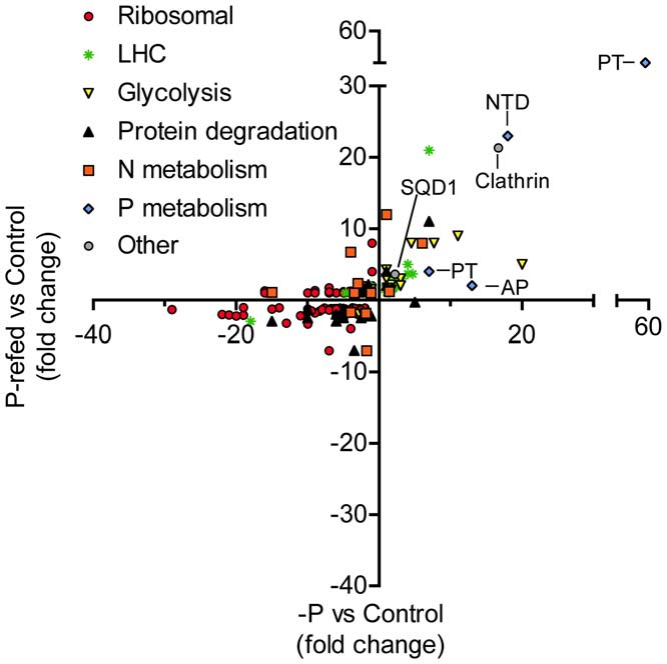
Abundances of specific categories of proteins. Scatter plot highlighting those proteins that fell into the categories of being putatively related to ribosomal, light harvesting complex-like (LHC), glycolysis, protein degradation, N-metabolism, P-metabolism, or other (e.g. clathrin). For those proteins involved in putative P-metabolism, specific proteins are highlighted and include: PT: Inorganic phosphate transporter, SQD1: Sulfolipid biosynthesis gene, NTD: 5′-nucleotidase, AP: Alkaline phosphatase. Clathrin is also noted. Fold-changes were calculated relative to the control treatment.

A number of proteins with known roles in N metabolism were most abundant in the replete control (Figure 2, Table S3). A urease enzyme (NCBI #: 323449776) was slightly less abundant in the −P treatment versus the control, although this result was not statistically significant (Figure 2, Table S3). However, in the P-refed treatment, it was significantly 7-fold less abundant (Figure 2, Table S3). Urease is an enzyme that breaks down urea into carbon dioxide and ammonia, and it is necessary for using urea as a potential N source. Urea and other organic N sources are thought to play an important role in forming and sustaining *A. anophagefferens* blooms [1], [22]–[24]. Also found in this cluster is a cyanase enzyme (NCBI #: 323447336). This cyanase was significantly less abundant under both −P and P-refed conditions (Table S3). Cyanases hydrolyze cyanate, a byproduct of urea breakdown, into ammonia and carbon dioxide and have been shown to be important for obtaining N from cyanate in cyanobacteria [25], [26]. Additionally, an ammonium transporter (NCBI #: 323457240) was found in this cluster and was over 4-fold less abundant in −P and almost 2-fold less abundant in P-refed conditions (Figure 2, Table S3). A transcript for this same ammonium transporter was up-regulated under N-deficient conditions [27]. In brief, these results suggest that the lower abundance of protein observed here is probably specific to P and not general nutrient stress. Finally, an acetamidase/formamidase (NCBI #: 323450867) is found in this cluster and is down-regulated 2.8-fold and 1.4-fold in the −P and P-refed treatments, respectively (Table S3). In the coccolithophore *Emiliania huxleyi*, it was demonstrated that activities of acetamidase and formamidase increased under N deficiency [28]. Transcriptome data showed an increase in an acetamidase/formamidase in *A. anophagefferens* under N-deficient conditions [13]. Again, as with the ammonium transporter, these results suggest that the acetamidase/formamidase is regulated differently by N and P deficiency. The lower abundance of these N-metabolism proteins in the −P treatment suggests that *A. anophagefferens* may reduce its N-scavenging machinery during P deficiency. The fact that these N-metabolism proteins are also low in the P-refed treatments suggests that once P deficiency is alleviated, the N-scavenging machinery takes longer than 24 hours to respond. These results could have implications for utilizing N metabolism/scavenging proteins as markers of N deficiency in field populations, given that their expression may also be indirectly controlled by P availability.

Finally, a selenoprotein was also relatively more abundant in the control treatment (Table S3). Enzymes containing selenium are often more catalytically active than similar enzymes lacking selenium [29], suggesting that a given metabolic function would require less selenoprotein relative to a non selenium-containing protein. The *A. anophagefferens* genome appears to be enriched in genes encoding possible selenoproteins and *A. anophagefferens* has a high selenium requirement [6]. Out of the 56 genes encoding selenoproteins [6], only two putative selenoproteins were detected in this study (NCBI #: 323452479 and 323451867), and the specific peptides containing selenoresidues were not identified in these proteins (Table S3). However, this is not evidence that putative selenoproteins are not important or do not contain selenium in this organism because, typically, methods that detect selenoproteins require the use of LC-ICP-MS verification and sample processing techniques designed to avoid Se residue destruction [30], [31]. Additionally, selenoproteins may be relatively low abundance and difficult to detect if less protein is needed due to higher catalytic efficiency. Selenoprotein 323451867 was significantly less abundant in the −P treatment compared to the control and P-refed treatments. If other selenoproteins are regulated by P availability, then the *A. anophagefferens* selenium quota may change with P supply. However, selenoprotein 323451979 was not abundant and did not show differential expression between these treatments (Table S3). The role of these selenoproteins in cellular metabolism is unknown, but tracking their abundances as a function of selenium and P availability would be an important area of future investigation.

### Highest abundance in P-refed

The 33 proteins in clusters C and E are most abundant in the P-refed treatment and of lower abundance in the −P and/or control treatments (Figure 1). These proteins are induced after phosphate is re-supplied to P-deficient cells. Many of these proteins were slightly more abundant in the −P condition relative to the control (Cluster E, Figure 1). It could be that these proteins are induced when phosphate is unavailable and continue to be produced even after phosphate is re-supplied. One of these proteins is a putative sulfolipid biosynthesis protein (SQD1) that is 2.1-fold more abundant under −P conditions (Figure 2, Table S3). In *Arabidopsis thaliana*, reduced phosphate availability increases SQD1 mRNA expression and protein product and leads to an increase in sulfolipid content [32]. In the ocean, it has been demonstrated that some phytoplankton are able to reduce their P requirement by substituting P lipids with sulfolipids [33]. The differential abundance of this sulfolipid biosynthesis protein (NCBI #: 323449174) suggests that *A. anophagefferens* employs a similar strategy of switching phospholipids for sulfolipids to adjust P quota. Analysis of the polar membrane lipids showed that cellular concentrations of the sulfolipid sulphoquinovosyldiacylglycerol (SQDG) was nearly 1.5-fold greater in the −P condition versus the control condition (2,864±29 versus 2,001±29 amol cell^−1^), while cellular phospholipids were approximately 8-fold less (133±11 versus 1,104±41 amol cell^−1^). In the P-refed condition, the putative sulfolipid biosynthesis protein was even more abundant (3.6 fold higher versus the control), than under the −P condition mentioned above (2.1- fold) meaning induction continues even after 24 hours of experiencing excess levels of phosphate. This result is unexpected because in the diatom *T. pseudonana*, P-deficient cells reduced their non-P lipids from ∼43% to ∼7% of their total lipid content over a period of 24 hours once phosphate became available [34], suggesting that 24 hours would be enough time to observe a change in abundance of proteins involved in this response. A delay in lipid replacement after P-addition could aid *A. anophagefferens* in maintaining lower P-quotas for longer time periods after nutrient pulses, perhaps conferring some advantage in their dynamic coastal environment where fluctuations between states of nutrient limitation could potentially be more rapid than in other areas. Additional work that examined lipids in concert with protein abundance in P-refed conditions would be useful to determine the timing and responses of lipids to P re-supply.

Similar to *T. pseudonana*, *A. anophagefferens* synthesizes the betaine lipid diacylglycerylcarboxyhydroxymethylcholine (DGCC) in response to P deficiency; concentrations of DGCC were 3,225±39 amol cell^−1^ under −P conditions but were undetectable under control conditions (<10 amol cell^−1^). Betaine lipids contain N, but do not contain P, therefore betaine lipids could represent a reasonable alternative to phospholipids, but their synthesis is contingent upon N availability. In this study, N was replete in all conditions. The protein responsible for synthesis of the betaine lipid diacylglyceryltrimethylhomoserine (DGTS) has been identified in the green alga *Chlamydomonas reinhardtii* (BTA1Cr) [35], but both the DGTS lipid and homologs of BTA1Cr are absent in *A. anophagefferens*. Very little is currently known of the synthesis of DGCC, although there is some evidence to suggest that, similar to DGTS, S-adenosyl methionine (SAM) is a key intermediate in its synthesis [36]. A SAM synthetase protein (NCBI #: 323448510) was detected in this study and was more abundant under −P and P-refed conditions relative to the control (Table S3). Given its pattern of abundance, this protein may be involved in DGCC synthesis. A time-course experiment that traced lipid composition in concert with SQD1 and enzymes that might be involved in DGCC synthesis would help elucidate these aspects of *A. anophagefferens* P physiology.

### Lowest abundance in control

There were 104 proteins generally more abundant in the −P and P-refed treatments relative to the control, falling into cluster F (Figure 1, Table S3). These proteins are more abundant when phosphate becomes deficient and continue to be present when P is resupplied. As such, proteins in this group are not actively degraded when phosphate is re-supplied to the −P cells and may continue to be produced. Within this cluster, 14 proteins are manually curated LHCs (Table S3). LHC proteins are known to have variable regulation patterns in other algae. For example diatom genes encoding LHC-like proteins were significantly up-regulated in iron starved conditions [37]. Additionally, a transcriptome profiling analysis in the coccolithophore *E. huxleyi* demonstrated down-regulation of an LHC-like protein during N starvation [38]. The 14 LHC-like proteins in this study were significantly more abundant in both the −P and P-refed treatments versus the control (Figure 2, Table S3). One of these 14 LHC-like proteins (NCBI# 323457207) mapped to a gene that was previously shown to be up-regulated at the transcriptional level under general N and P stress [13]. It is difficult to predict whether these LHC proteins are involved in light harvesting, photoprotection, or some other physiological role and a more detailed study that quantifies RNA levels and protein levels in a variety of stress conditions would be needed to discern the variables governing LHC expression patterns.

Also in this cluster were 9 proteins involved in glycolysis, including a phosphoglucomutase (NCBI#: 323452848), phosphoglucose isomerase (NCBI#: 323455682), a triose phosphate isomerase (NCBI#: 323447110), a glyceraldehyde-3-phosphate dehydrogenase (NCBI#: 323449032), an enolase (NCBI#: 323453907), a pyruvate kinase (NCBI#: 323450876), a UTP–glucose-1-phosphate uridylyltransferase (NCBI#: 323452847), a nucleoside diphosphate kinase (NCBI#: 323454769), and a phosphoenolpyruvate carboxylase (PEPC) (NCBI#: 323453325) (Figure 2, Table S3). Glycolysis is the conversion of one molecule of glucose into two molecules of pyruvate, and requires 2 molecules of inorganic phosphate. Due to this P requirement, glycolysis enzyme activities in higher plants are affected by P supply in order to bypass those reactions that demand phosphate (see review: [39]). Based upon the abundance patterns of these nine enzymes under −P and P-refed conditions, *A. anophagefferens* also appears to modulate or switch its glycolytic pathway in response to P supply. For example, PEPC can serve as a glycolytic bypass enzyme by diverting phosphoenolpyruvate (PEP) to oxaloacetate (OAA) and releasing inorganic phosphate. This bypass has been suggested in higher plants [40] and the green alga *Selenastrum minutum* [41]. OAA can then be converted to malate through the activity of malate dehydrogenase and eventually to pyruvate through a malic enzyme, thus completing the bypass of the ADP-requiring step of converting PEP directly to pyruvate catalyzed by pyruvate kinase [39]. However, considering that two PKs (see below) were more abundant during −P conditions and no malic enzyme was detected in this study, it is difficult to interpret whether *A. anophagefferens* is using PEPC to bypass the ADP-requiring PK step of glycolysis, or simply liberating inorganic phosphate from PEP and replenishing tricarboxylic acid cycle intermediates.

Other proteins in the glycolysis pathway did not show differences in abundance among the three treatments (Table S2) while some showed differences, but did not fall into this particular cluster. For example, another pyruvate kinase (NCBI#: 323453799) was 20-fold more abundant under −P relative to the control, but only 5-fold more abundant in P-refed conditions (Figure 2, Table S3), suggesting a stronger response to P re-supply relative to other glycolysis enzymes. Another glyceraldehyde-3-phosphate dehydrogenase (NCBI#: 323455041) showed lowest abundance under −P and P-refed conditions, but highest abundances in the control treatment (Figure 2, Table S3). These results reflect the complexity of how *A. anophagefferens* is tailoring its glycolysis pathway to conserve P, while still trying to meet its respiration demands.

A number of proteins with known roles in P metabolism are found in cluster F, with lowest abundance in the control (Figure 1, Table S3). Two inorganic phosphate transporters are significantly more abundant in both the −P and P-refed treatments (Figure 2, Table S3). One of the phosphate transporters (NCBI #: 323454760) is 59-fold more abundant in the −P treatment and 50-fold more abundant in the P-refed treatment compared to the control, while the other phosphate transporter (NCBI #: 323456737) is 7-fold more abundant in the −P treatment and 4-fold in the P-refed treatment) (Figure 2, Table S3). This suggests that *A. anophagefferens* makes more phosphate transporters under P deficiency. Other eukaryotic algae have also been observed to employ this same strategy [38], [42]. In the P-refed condition, these phosphate transporters are lower than −P, but are still elevated relative to the control (Figure 2, Table S3). This is evidence of a lag between environmental changes and protein response and demonstrates that 24 hours is not enough time to observe a significant decrease in these phosphate transporters, possibly because these membrane proteins are not actively degraded.

**Figure 3:**
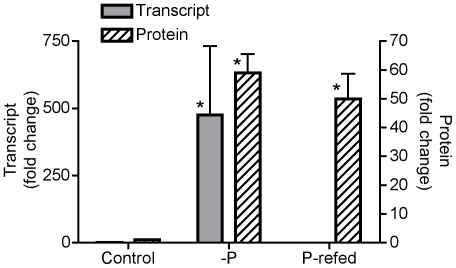
Transcript and protein abundances of a phosphate transporter. Bar graph comparing the expression of one inorganic phosphate transporter (NCBI #: 323454760) at the transcriptional level and abundance at the protein level under control, −P and P-refed conditions. Transcript data are plotted as fold change relative to the control condition using the comparative C_T_ method for qRT-PCR with a reference gene that encodes a ubiquitin-conjugating enzyme. Error bars for transcript data specify standard error of the average fold change of triplicate measurements on a single biological replicate between the sample condition (control, −P, P-refed) and the reference condition (control). Protein data are plotted as fold change relative to the control condition based upon spectral counts. Error bars for protein data specify standard error of the fold change among triplicate technical measurements of spectral counts for each condition. An asterisk (*) indicates that the fold change was significantly higher than the reference condition (*p*-value<0.001) based upon a pair-wise fixed reallocation randomization analysis for the transcript data and a Fisher exact test for the protein data.

To further explore turnover, an additional experiment was performed to re-create control, −P, and P-refed conditions and test whether or not expression of the more abundant phosphate transporter (NCBI #: 323454760) changed at the transcriptional level over this 24 hour period (Figure 3). The phosphate transporter transcript is significantly up-regulated (*p*-value<0.001) over 400-fold under −P conditions relative to the control (Figure 3). After 24 hours of being re-fed phosphate, the transcript expression levels of the phosphate transporter were not detected (Figure 3). A biological replicate was examined for each condition and the results were similar with the phosphate transporter being significantly up-regulated (*p*-value<0.001) over 500-fold under −P and not detectable under P-refed conditions. In both P-refed biological replicates, the reference gene amplified in an efficient C_T_ range, but the phosphate transporter did not amplify, indicating that the phosphate transporter transcript abundance was too low to detect, but the RNA and subsequent cDNA were of good quality. In the specific case of this phosphate transporter, transcript abundance declines rapidly in 24 hours, while the protein abundance appears to decline more slowly. Consequently, the interpretation of transcript and protein abundance measurements for this transporter should consider these timing differences, where the transcript could detect short-term P supply, and the protein would reflect the cell's physiological history as well as its current environment. While the slower decline of the phosphate transporter protein relative to its transcript may be due to the slower degradation of proteins associated with membranes [43], it is also possible that there has been little selection pressure to actively degrade this transporter versus allowing it to dilute away with growth and cell division. The observed persistence of the phosphate transporter for more than 24 hours after re-exposure to P would allow the cell to replenish its depleted phosphate cellular quota and may be particularly advantageous in an environment of variable P supply.

A 5′-nucleotidase (NCBI #: 323455642) was also significantly more abundant in both −P (18-fold) and P-refed (23-fold) conditions versus the control (Figure 2, Table S3). 5′-nucleotidase enzymes cleave the phosphate group from the 5′ end of the sugar moiety in nucleotides and may be used by algae to scavenge phosphate from exogenous nucleotides in the environment [44], [45]. Consistent with an extracellular function, SignalP (version 3.0) was used to determine that this 5′-nucleotidase contains a signal peptide suggesting this protein is secreted [46], [47]. Nucleotides released from grazing or cell lysis could potentially be a reservoir for P in the ocean with concentrations reaching 10–20 nM [48]. *A. anophagefferens* can utilize adenosine monophosphate (AMP) as a sole P source in culture [13]. These data, combined with the 5′-nucleotidase protein data in this study, suggests that nucleotides may be an important source of P for *A. anophagefferens* when DIP is scarce. As with the phosphate transporter proteins, the abundance of 5′-nucleotidase did not decline when cells were re-fed with P suggesting that the 5′-nucleotidase protein is not actively degraded upon P addition.

Finally, a clathrin protein (NCBI #: 323455486) was found in this cluster and was over 16-fold more abundant in −P and 21-fold more abundant in the P-refed conditions versus the control (Figure 2, Table S3). Recently, clathrin was shown to be one of the most abundant proteins in the diatom *T. pseudonana* [17] and was also detected in a proteomic analysis of the coccolithophore *E. huxleyi* [49]. Here, clathrin in *A. anophagefferens* was not only abundant, but was variable with higher abundances in the −P and P-refed treatments relative to the control. Clathrin is the major coat protein of clathrin-coated vesicles (CCVs) [50]. CCVs selectively sort and transport proteins and lipids from the outer membrane of cells to endosomes (see [51], [52] for reviews of CCV formation and function). Clathrin-mediated endocytosis (CME) is also a mechanism by which eukaryotic cells can internalize nutrients and other macromolecules [53]. Given that CME can be a mechanism for internalizing nutrients, this protein could play a direct role in P scavenging from the environment. Alternatively, perhaps clathrin is involved in reconfiguring the lipid composition of cellular membranes since *A. anophagefferens* decreases phospholipids and increases non-phospholipids under −P conditions. The fact that clathrin has been shown to be abundant in diatoms, coccolithophores, and now the pelagophyte *A. anophagefferens* is intriguing and warrants further investigation [17], [49].

### Highest abundance in −P

There were 26 unique proteins that were most abundant under −P conditions and fall into clusters G and H (Figure 1, Table S3). These proteins are most abundant under P deficiency, but are rapidly turned over 24 hours after being refed phosphate. Four of these proteins are LHCs and their presence is consistent with the observation that LHC proteins in *A. anophagefferens* are induced during nutrient stress (Figure 2, Table S3). Proteins with known roles in P metabolism found in this cluster include an alkaline phosphatase (NCBI #: 323455998) which increased 4.3-fold in −P versus control, and was not significantly different in the P-refed versus control (Figure 2, Table S3). Alkaline phosphatases cleave phosphate from a variety of organic molecules and are induced in other algae during P deficiency [24], [45], [54]. The induction of this alkaline phosphatase during P-deficient conditions suggests *A. anophagefferens* has the ability to utilize phosphoesters to meet its P demand when DIP is unavailable. Furthermore, the induction of alkaline phosphatase under −P conditions, combined with the results from the 5′-nucleotidase discussed above, is consistent with the observation that at peak cell densities during *A. anophagefferens* blooms there is a significant drawdown of DOP [11]. After 24 hours of being re-fed P, the abundance of the alkaline phosphatase is similar to the control, suggesting rapid turnover or degradation of this protein upon release from P deficiency. This result is similar to findings from the coccolithophore, *E. huxleyi*, where alkaline phosphatase activity was induced under P-deficient conditions, and this activity rapidly decreased 24 hours after cells were refed P [45]. This is in contrast to the P scavenging proteins that remain abundant in P-refed conditions (e.g. inorganic phosphate transporter and 5′-nucleotidase). Alkaline phosphatase has been observed to be prone to loss from *E. coli* and a marine cyanobacterium [55], and thus may be rapidly lost from the cell rather than being targeted specifically for degradation. Regardless, the disparities in protein presence and abundance among different P-deficient induced proteins after release from P deficiency are intriguing, and should be considered for interpreting protein presence and abundance in natural populations under conditions of non-steady state phosphate and DOP concentrations. In this case, the phosphate transporter and alkaline phosphatase would be indicative of P deficiency at different timescales.

### Lowest abundance in −P

The 80 proteins in cluster D are most abundant in the control and P-refed treatments and low abundance in the −P treatment (Figure 1, Table S3). There are a few N-related proteins in this cluster, including a nitrate transporter (NCBI #: 323448256), a nitrate and nitrite reductase (NCBI #: 323453433 and 323453434) and a urea transporter (NCBI #: 323451781) (Figure 2, Table S3). The down-regulation of these proteins under −P conditions is consistent with the N proteins discussed above. However, these proteins appear to be more responsive than those discussed earlier as they are relatively abundant again under P-refed conditions.

The majority of the proteins in this category are ribosomal (Figure 2, Table S3). Ribosomes are formed from ribosomal proteins along with ribosomal RNA, and are the macromolecular machines responsible for translation and protein syntheisis. Protein synthesis requires a large energy input. For example, up to 40% of *E. coli's* total cell energy turnover goes toward protein synthesis [56]. Therefore, protein synthesis must be tightly controlled to meet the biosynthetic demands of the cell and not waste resources on unnecessary protein synthesis. In *A. anophagefferens* there is a global down-regulation of ribosomal proteins during P deficiency. It is unclear whether this is a strategy to conserve resources, or a by-product of stationary growth. Once phosphate is available, these ribosomal proteins are immediately abundant again, suggesting that they are tightly coupled to the cell's growth environment and are indicative of nutrient availability to *A. anophagefferens*.

### Insights gained from P resupply

Some P-responsive proteins decreased in abundance upon P resupply while others did not. This is likely a function of how quickly these proteins are degraded after P becomes abundant. The variability in this turnover may be a function of the position of the protein within the cell, for example integral membrane proteins may be degraded slowly since they are more difficult to access. Another explanation is that this time could also be a function of the protein's continued utility to the cell upon P resupply. Perhaps upon P addition it is advantageous to keep phosphate transporters in abundance for some time to take full advantage of the sudden increase. In contrast, alkaline phosphatase is no longer of utility once there is plenty of inorganic P available, and so this protein is quickly degraded.

One of the primary aims of these types of studies is identifying genes and proteins that can be used as biomarkers of nutritional physiology in field populations. This study highlights the importance of including a refed treatment in such analyses. A simple +P/−P only gives a snapshot of protein abundances. For example, both the phosphate transporter and alkaline phosphatase proteins were more abundant under −P conditions relative to the control. Without a P-refed treatment, both proteins would be considered equally good biomarkers for P deficiency. However, this study revealed that due to differences in protein turnover, these two proteins could provide information about different stages of P deficiency under non-steady state nutrient conditions such as during a bloom situation.

### Proteome/transcriptome comparison

**Figure 4:**
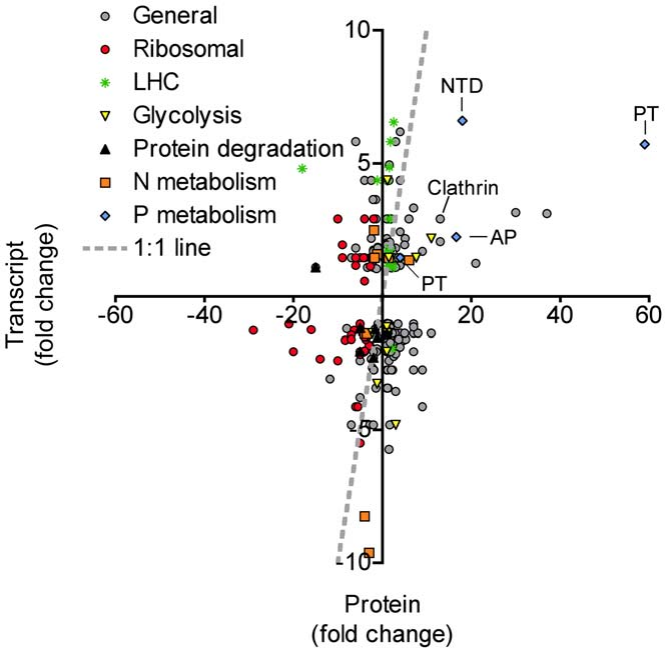
Protein versus transcript abundances. Scatter plot comparing the proteome data and transcriptome data in the −P treatment. All fold-changes are calculated relative to a control. The gray dashed line represents the 1∶1 line. Data points falling on or near that line have similar regulation patterns at both the transcript and protein level. Specific protein IDs pointed out include: Clathrin, PT: Inorganic phosphate transporter, NTD: 5′-nucleotidase, and AP: Alkaline phosphatase. The sulfolipid biosynthesis protein (SQD1) was not represented in the transcriptome data.

A previous study examined the transcriptome of *A. anophagefferens* under nutrient replete (control) and −P conditions using Long Serial Analysis of Gene Expression (Long-SAGE) [13]. The transcriptome and proteome data were compared to examine choreography between the two datasets. Of the 641 unique proteins in this study, 257 were also present in the transcriptome (Table S4). An examination of the −P relative to control fold-change for both the transcript data (SAGE tag counts) and protein data (average abundance score) indicate that for some targets, the transcriptome and proteome responses are coordinated (Figure 4, Table S4). The inorganic phosphate transporter (NCBI #: 323454760) and alkaline phosphatase (NCBI #: 323455998) display significant up-regulation at both the transcript and protein levels (Figure 4). Less tightly linked, but still up-regulated in the −P treatment at both the transcript and protein levels are a 5′-nucleotidase (NCBI #: 323455642) and clathrin (NCBI #: 323455486) (Figure 4). No transcript data could be found for the sulfolipid biosynthesis protein from the Long-SAGE study [13]. Long-SAGE tags are generated at the most 3′ *NLAIII* site of an mRNA and are often found in the untranslated region (UTR) of an mRNA. The genome was searched in the 3′ direction of the sulfolipid biosynthesis gene and no tag was found. A higher resolution (deeper sequencing) analysis or targeted gene expression assay would be needed to determine how the transcript is for this sulfolipid biosynthesis gene is regulated.

N-metabolism and LHC genes show little correlation in expression patterns between transcript and protein levels (Figure 4). Ribosomal proteins tend to be ubiquitously down-regulated under −P at the protein level, and for the most part, at the transcript level as well (Figure 4). Genes involved in protein degradation also appear to be somewhat choreographed with expression patterns at both the transcript and protein level indicating down-regulation under −P conditions (Figure 4, Table S4). This suggests that certain proteins are rapidly being turned over under nutrient replete conditions where growth rates are high. With the data available here, it is unclear as to which specific proteins are being targeted, and therefore difficult to put the expression patterns in context of adapting to P deficiency. Nonetheless, in order for an organism to change its proteome to adapt to variations in its environment, new proteins have to be made and proteins which are no longer needed must be recycled, and given the extensive proteome rearrangement observed here in response to P supply, it is not surprising that genes involved in protein degradation are also sensitive to P supply.

Although the actual fold changes are quite different between the transcripts and proteins for a given gene, 27.2 percent of the genes showed a “correlated” pattern (see methods). Approximately 58.4 percent of genes were considered “neutral”, meaning the fold changes for either the transcript, protein, or both were less than 1.5 fold different from the control (Figure 4, Table S2). The patterns displayed by these “neutral” genes could partly be explained if there is a lag between the induction of transcripts and subsequent translation of proteins (e.g. high transcript, neutral protein) or the repression of transcripts and turnover of proteins (e.g. neutral transcript and high protein). In yeast it has recently been shown that transcriptional patterns 1–2 hours after treatment were best correlated with protein abundances 4–6 hours after treatment with the antibiotic rapamycin, supporting the idea of a lag between induction of transcripts and translation of proteins [57]. Furthermore, in yeast it was recently reported that an induction of mRNA due to osmotic stress is well correlated with an induction of proteins, but transcript reduction produced almost no change in the corresponding proteins [58]. Clearly, a snap shot view of the transcriptome and proteome at the same time point would not give the most correlated pattern because the transcripts and proteins are being induced and degraded at different time scales. Only 14.4 percent of genes showed a “not correlated” pattern, where the transcript and protein fold changes were opposite. This result could be due to the transcript and protein data being generated from different biological samples, where slight variations in growth rate and point of harvest within the diel cycle could make a large difference in the expression patterns of certain genes.

The relative timing of the transcriptional and protein responses is biologically interesting and could be practically useful in interpreting expression patterns of both transcripts and proteins from environmental datasets. From culture studies, the expression patterns of certain genes can be linked to a cell's physiological condition. For example, the phosphate transporter discussed in this study is significantly up-regulated at both the transcript and protein level when *A. anophagefferens* experiences P deficiency. This gene could thus be used as a marker for examining P deficiency in natural populations. However, the abundance of the protein may have a different interpretation than the abundance of the transcript. In this example, the phosphate transporter protein was still abundant after the cells were exposed to replete P, and its presence may indicate P deficiency in the recent past and not necessarily the cell's current environment. The transcript for this phosphate transporter appeared to give finer resolution for assaying P deficiency, and its abundance may be more indicative of the cell's current geochemical environment. Conversely, since some genes are not being correlated, the abundance of a transcript may not equate to the protein being abundant and it would be difficult to infer activity, in a strictly temporal sense, based upon transcript abundance alone. These issues should be kept in mind when working with microbial community, metatranscriptomic, or metaproteomic datasets.

### Conclusion

This study examined the timing of global protein responses in algal cells subjected to, and then alleviated from, P deficiency. Throughout this study, a number of proteins were identified as being differentially regulated by P availability. *A. anophagefferens* increases its ability to scavenge or conserve P by: (1) up-regulating proteins involved in DOP acquisition, such as a 5′-nucleotidase and alkaline phosphatase; (2) increasing its ability to transport phosphate by up-regulating more phosphate transporters or switching to a more efficient phosphate transporter; (3) lowering its P demand by switching sulfolipids for phospholipids; (4) and adjusting its glycolysis pathway. Insight into the timing of these responses was gained by examining protein abundances in a P-refed condition. In this case, many proteins were more abundant under P deficiency, but were not repressed 24 hours after being refed phosphate. This lag in response provides insight into the biological response to P deficiency, as well as the evolved coordination between transcript and protein expression. In addition, this lag has practical importance in the use of transcript and protein abundances as indicators of physiological state (e.g. P stress) *in situ*. If P acquisition proteins, like the phosphate transporter that is not quickly degraded, are abundant in a field sample, it may not be entirely reflective of the immediate P abundances in the environment in dynamic non-steady state bloom conditions. Instead, it may be reflecting a previous environmental condition, or multiple different conditions integrated over time. These considerations are important for interpreting transcriptomic and proteomic profiles in metadatasets, particularly in relation to nutrient abundances. A comparison with the transcriptome shows that P-responsive proteins related to P metabolism/scavenging appear to be correlated. A time lag between the transcriptional responses versus the protein responses may account for those genes that are “neutral” or “not correlated”. Finally, the breadth of response at both the transcriptome and proteome level of *A. anophagefferens* to P deficiency, combined with field observations of significant DOP drawdown during peak cell densities, suggest that P may play a more important role in brown tide formation, persistence and decline than previously thought.

## Materials and Methods

### Culture conditions

An axenic culture of *A. anophagefferens* strain CCMP 1984 was obtained from the Provasoli-Guillard Center for the Culture of Marine Phytoplankton (CCMP). Culture treatments were grown in triplicate Fernbach flasks in 2L of media per replicate at 18°C on a 14 hour∶10 hour light∶dark cycle at 150 µmol quanta m^−2^ s^−1^. Locally collected Vineyard Sound seawater was filtered (0.2 µm) and used to make modified L1 media with no added silica [59]. No specific permits were required for the collection and use of this seawater. P concentrations were modified as follows: 36 µM phosphate for the control treatment and 1 µM phosphate for the P-deficient (−P) treatment. Vitamins (thiamine, biotin, and B_12_) were sterile filtered and added after autoclaving. Each flask was then inoculated with *A. anophagefferens* stock culture to a starting concentration of 10^5^ cells mL^−1^. Growth was monitored daily by cell counts on a hemacytometer and relative fluorescence using a Turner Designs fluorometer. Cells were harvested by centrifugation to form pellets and immediately stored in LN. Control treatment cells were harvested on day 6 during exponential phase of growth and −P treatment cells were harvested on day 8, at onset of stationary phase (Figure S2). Phosphate was then added back to the remaining −P cells to a final concentration of 36 µM. These P-refed cells were harvested 24 hours later.

### Protein extraction and digestion

Cell pellets (single biological replicate from each treatment) were resuspended in 700 µL B-PER reagent (Thermo Scientific, Rockford, IL) supplemented with 5 mM EDTA and 1 mM phenylmethanesulfonylfluoride (a serine protease inhibitor). Samples were incubated at room temperature for 40 minutes with occasional gentle vortexing and then incubated on ice for 10 minutes. The cells were then sonicated with a microtip (Branson digital sonifier) on ice for 1 minute at constant duty cycle. Samples were centrifuged for 40 minutes at 14,100 RCF and 4°C, and protein was precipitated out of the supernatants overnight in 50% acetone 50% methanol 0.5 mM HCl at −20°C. Precipitated protein was collected by centrifugation at 14,100 RCF for 30 minutes at 4°C and dried by speed vacuum at room temperature. Protein was resuspended in 100 uL of the extraction buffer. Aliquots were taken for protein determination by DC assay using bovine serum albumin as a protein standard (BioRad Inc., Hercules CA). Proteins were stored at −80°C until digestion.

Protein samples were digested following the tube gel digestion procedure [60] with minor modifications. Briefly, samples were immobilized in 15% acrylamide in pH 7.5 Tris buffer, fixed with 10% acetic acid and 50% ethanol, washed successively with 10% acetic acid and 50% methanol, then acetonitrile and 25 mM ammonium bicarbonate to remove detergents and protease inhibitors and then cut into 1 mm^3^ pieces. Samples were reduced with 10 mM dithiothreitol (DTT) at 56°C for 1 hour, alkyated with 30 mM iodoacetamide for 1 hour, and then washed in 25 mM ammonium bicarbonate and digested with trypsin in 25 mM ammonium bicarbonate for 16 hours at 37°C (1∶20 ratio trypsin to total protein, Promega Gold Mass Spectrometry Grade, Promega Inc., Madison WI). The peptides were extracted by three successive additions of 50% acetonitrile (Fisher Optima) with 5% formic acid (Michrom Ultra Pure). The extracted peptides were combined and concentrated by speed vacuum for about three hours to less than 20 µL, diluted with 2% acetonitrile and 0.1% formic acid in water (Fisher Optima) and stored at −80°C.

### Shotgun mass spectrometry

The protein digestions were analyzed (4 ug total protein per analysis) using a peptide Cap Trap in-line with a reversed phase Magic C18 AQ column (0.2×150 mm, 3 µm particle size, 200 Å pore size, Michrom Bioresources Inc. Auburn CA) on a Paradigm MS4 HPLC system (Michrom Bioresources Inc.) at a flow rate of 2 µl minute^−1^, similar to previously described methods [24]. A LTQ linear ion trap mass spectrometer (Thermo Scientific Inc. San Jose CA) was used with an ADVANCE nanocapillary captive electrospray source (Michrom Bioresources Inc.). The chromatography consisted of a hyperbolic gradient from 5% buffer A to 95% buffer B for 300 minutes, where A was 0.1% formic acid (Michrom Ultra Pure) in water (Fisher Optima) and B was 0.1% formic acid in acetonitrile (Fisher Optima). The mass spectrometer was set to perform MS/MS on the top 7 most abundant ions using data-dependent settings with a dynamic exclusion window of 30 seconds. Ions were monitored over the range of 400–2000 m/z. Technical triplicate measurements were conducted for each biological sample. Technical replicates of spectral count data from control conditions were plotted against each other to ensure accuracy of the method (Figure S3).

### Mass spectrometry data processing and proteome profiling

The mass spectra collected in this study were searched using SEQUEST (Bioworks version 3.3, Thermo Inc., San Jose CA). An amino acid database for *A. anophagefferens* was constructed by combining all “project data” from the *A. anophagefferens* genome sequencing (11520 sequences from NCBI: http://www.ncbi.nlm.nih.gov/genomeprj/13500) and adding plastid proteins (105 sequences from NCBI: http://www.ncbi.nlm.nih.gov/genomeprj/36625), along with common contaminants as well as a reversed ‘decoy’ version of these databases for false discovery rate analysis (data downloaded on March 8th, 2011). Searches were conducted with a static modification for cysteine of +57 for alkylation by iodoacetamide and allowing for variable modifications expected if methionine was oxidized (+16), if cysteine or methionine were present as seleno-residues (+47) or if selenocysteine was modified to dehydroalanine (−91) [30]. Database search results were further processed using the PeptideProphet statistical model [61] within Scaffold 3.0 (Proteome Software Inc., Portland OR). At least two peptides had to map to a protein sequence to be included in the data. Relative protein abundance was determined using spectral counting in Scaffold 3.0. Spectral counts are normalized across samples in each experiment, including technical replicates, to allow comparison of relative protein abundance and result in a quantitative value abundance score, as previously described [19]. Proteins discussed as ‘differentially abundant’ were determined by the Fisher exact test as previously described [62] with *p*-values<0.05. A complete list of *p*-values for all proteins can be found in Table S2. False discovery identification rate was estimated using a reversed decoy database as previously described [63].

The proteins that met the criteria for being differentially abundant were compared by a hierarchical cluster analysis using Cluster 3.0 [64]. Average abundance scores for each sample were log transformed, centered about the mean and normalized by multiplying all values by a scale factor S so that the sum of the squares of the values for each protein is 1.0. The treatments were not centered or normalized. The data were then clustered by both protein and treatment using a centered correlation as metric and complete linkage as clustering method. The data were displayed using Java Tree View [65].

### Proteome comparison to transcriptome

A previous study [13] generated transcriptome expression data under conditions identical to those examined in this study, excluding the P-refed cells, using Long Serial Analysis of Gene Expression (Long-SAGE). Tag data from Long-SAGE were compared to the protein data obtained from this study. Only the −P and control treatments, and only genes with products identified in this study as well as the Long-SAGE study with at least two tags mapping to a given protein ID, were included in this analysis. Abundance scores from the proteome and tag counts from the transcriptome were compared using fold change in the −P treatment relative to the control. If the fold change resulted in a fraction due to a higher abundance in the control versus the −P, then the negative inverse was taken (e.g. a fold change of 0.5 would be converted to −2). To quantify the percentages of genes that were correlated at the transcript and protein level fold changes were compared between the transcript and protein data. If the transcript and protein data both showed a fold change ≥1.5 or ≤−1.5, that gene was considered correlated. If the transcript showed a fold change ≥1.5 and the protein showed a fold change ≤−1.5, or vice versa, that gene was considered not correlated. If either the protein or transcript showed a fold change between −1.5 and 1.5, that gene was considered neutral.

### Targeted gene expression

A follow-up experiment was conducted to examine targeted gene expression of an inorganic phosphate transporter (NCBI #: 323454760). Control, −P, and P-refed conditions were generated as discussed above. Cells were collected on a 0.2 µm polycarbonate filter by vacuum filtration and immediately placed in CTAB extraction solution (Teknova, Hollister CA) amended by the addition of 1% mass/volume polyvinylpyrrolidone. Samples were stored at −80°C until further processing.

Total RNA was isolated from each sample using the UltraClean® Plant RNA Isolation Kit (MO BIO Laboratories, Inc., Carlsbad CA) using modified manufacturer's instructions. First, samples were centrifuged at 10,000× g to separate cell lysate from the filter and 650 uL of sample was transferred to a fresh 1.5 mL tube. Secondly, 300 µL of PMR1 was added to each sample and mixed by vortexing followed by the addition of 800 µL of PMR4 to each sample and again mixed by vortexing. Finally, samples were loaded onto the columns and RNA extraction continued according to manufacturer's instructions. Isolated RNA was then treated with TURBO™ DNase (Ambion, Austin TX) to remove potential genomic DNA contamination and RNA was then quantified spectrophotometrically. A total of 100 ng of RNA was primed with oligo dT primers and reverse transcribed into cDNA using the iScript Select cDNA Synthesis kit (Bio-Rad, Hercules CA). For each sample, a second reaction was performed in which no reverse transcriptase was added to serve as a control for genomic DNA contamination in subsequent analysis. These controls were all negative suggesting no contamination.

Species-specific primers were designed from genomic sequences using MacVector (MacVector, Inc., Cary NC). Amplicons were screened for secondary structure using Mfold software [66] to confirm the primers were qPCR compatible. A qRT-PCR assay was designed to optimize primer efficiency and examine relative abundance of cDNA transcripts across treatments using the comparative C_T_ method [67]. All qRT-PCR reactions were run in triplicate using Brilliant® II Fast SYBR® Green qRT-PCR Master Mix (Agilent Technologies, Santa Clara CA) and analyzed on a Bio-Rad iCycleriQ® qRT-PCR detection system (Bio-Rad. Hercules CA) with the following cycling parameters: 1× 95°C 5 minutes, 45×: 95°C for 10 seconds, 62°C for 30 seconds. A dissociation curve was performed to check for non-specific amplification by holding PCR reactions at 95°C for 1 minute and lowering the temperature by 0.5°C every 10 seconds to 55°C. The ΔC_T_ (C_T_ target – C_T_ reference) was examined over a range of cDNA concentrations to ensure equal amplification efficiencies between target and reference amplicons. A plot of the log_10_ cDNA dilution versus ΔC_T_ was constructed to ensure the value of the slope did not differ significantly from zero. In this case, a constitutively expressed gene encoding an *A. anophagefferens* ubiquitin-conjugating enzyme (UbE2) was used as a reference gene [27]. For UbE2, primer sequences are 5′: GCGAGCTCCAGGACTTTATG and 3′: CGGGGTCGAGGAAGTAGAC with an amplification efficiency of 102.7% at a forward and reverse primer concentration of 300 nM and amplicon size of 192 nucleotides. For the phosphate transporter, primer sequences are 5′: CATCCTCTACGGCATCACCAAG and 3′: ATCCAGAAGACGGAGTTGACGC with an amplification efficiency of 104.9% at a forward and reverse primer concentration of 300 nM and 141 nucleotide amplicon size. Here, the reference condition was P-replete grown cells, the reference gene was UbE2, and the experimental treatments were −P grown cells and P-refed cells. All template (cDNA) concentrations were 0.1 ng in the qRT-PCR reactions. Fold-change was determined using the Relative Expression Software Tool (REST) located at http://www.gene-quantification.de/download.html. REST accounts for differences in efficiency between primer sets when calculating fold changes. The *p*-values were determined by a pair-wise fixed reallocation randomization analysis [68].

### Polar membrane lipid analysis

The polar membrane lipid compositions of *A. anophagefferens* were examined using previously described approaches [34], [69]. Briefly, batch cultures of *A. anophagefferens* strain CCMP 1984 were grown in either control or −P media as described above. Cells were harvested in log phase by filtration on GF/F filters, and immediately frozen in liquid N. Polar lipids were later extracted into dichloromethane [34] and analyzed by HPLC/MS/MS using normal phase chromatographic conditions on an Agilent 1200 HPLC coupled via an electrospray ionization source to a Thermo Vantage TSQ triple quadrupole mass spectrometer [69].

## Supporting Information

Figure S1 **Protein abundances in each treatment.** Scatter plot with the abundance of each protein in the (A) −P and control conditions and (B) P-refed and control conditions. Blue squares indicate proteins that are significantly different (*p*-value<0.05) between the conditions based upon the Fisher exact test. Red triangles specify proteins that are greater than 2-fold different between conditions. The gray dashed line indicates equal abundances between the conditions.
(TIFF)

Figure S2 **Growth under experimental conditions.** Growth of *A. anophagefferens* under nutrient replete (control) and P-deficient (−P) conditions plotted as Relative Fluorescence Units. The control treatment was harvested on day 6. On day 8, −P cells were harvested to generate the −P treatment. Remaining −P cells were re-fed phosphate and harvested 24 hours later to generate the P-refed treatment.
(TIFF)

Figure S3 **Spectral counting accuracy.** Scatter plot demonstrating the precision of the method for spectral counting. Technical replicates of spectral count data from control conditions are plotted against each other. A 1∶1 line is shown for comparison.
(TIFF)

Table S1 **Peptides.** Peptide identifications.
(XLSX)

Table S2 **Annotated proteins.** Annotated proteins identified in this study. N/A signifies a fold change could not be calculated because the average spectral count in the control treatment had a value of zero. No ID means that the protein is not represented in the Joint Genome Institute's (JGI) predicted protein dataset from the genome sequence.
(XLSX)

Table S3 **Differentially abundant proteins.** Proteins separated by cluster. Proteins listed in order of how they appear in Figure 2 from top to bottom. Fold changes were calculated by dividing the treatment (−P or P-refed) by the control. If the average spectral count in the control was zero, then it was coverted to 0.333 which represents the average spectral count if there was one count in one of the three technical replicates. This was done to avoid division by zero. If the fold change resulted in a decimal, the negative inverse was taken (e.g. 0.5 would be converted to −2).
(XLSX)

Table S4 **Protein and transcript data.** Proteins and associated transcript data (SAGE tag counts). Fold changes were calculated as described in Table S3.
(XLSX)
