# Supplementary figures and images for: Proteome Changes Driven by Phosphorus Deficiency and Recovery in the Brown Tide-Forming Alga Aureococcus anophagefferens
Source: PLoS One. 2011 Dec 14;6(12):e28949. doi: 10.1371/journal.pone.0028949 (PMC3237563; doi:10.1371/journal.pone.0028949)

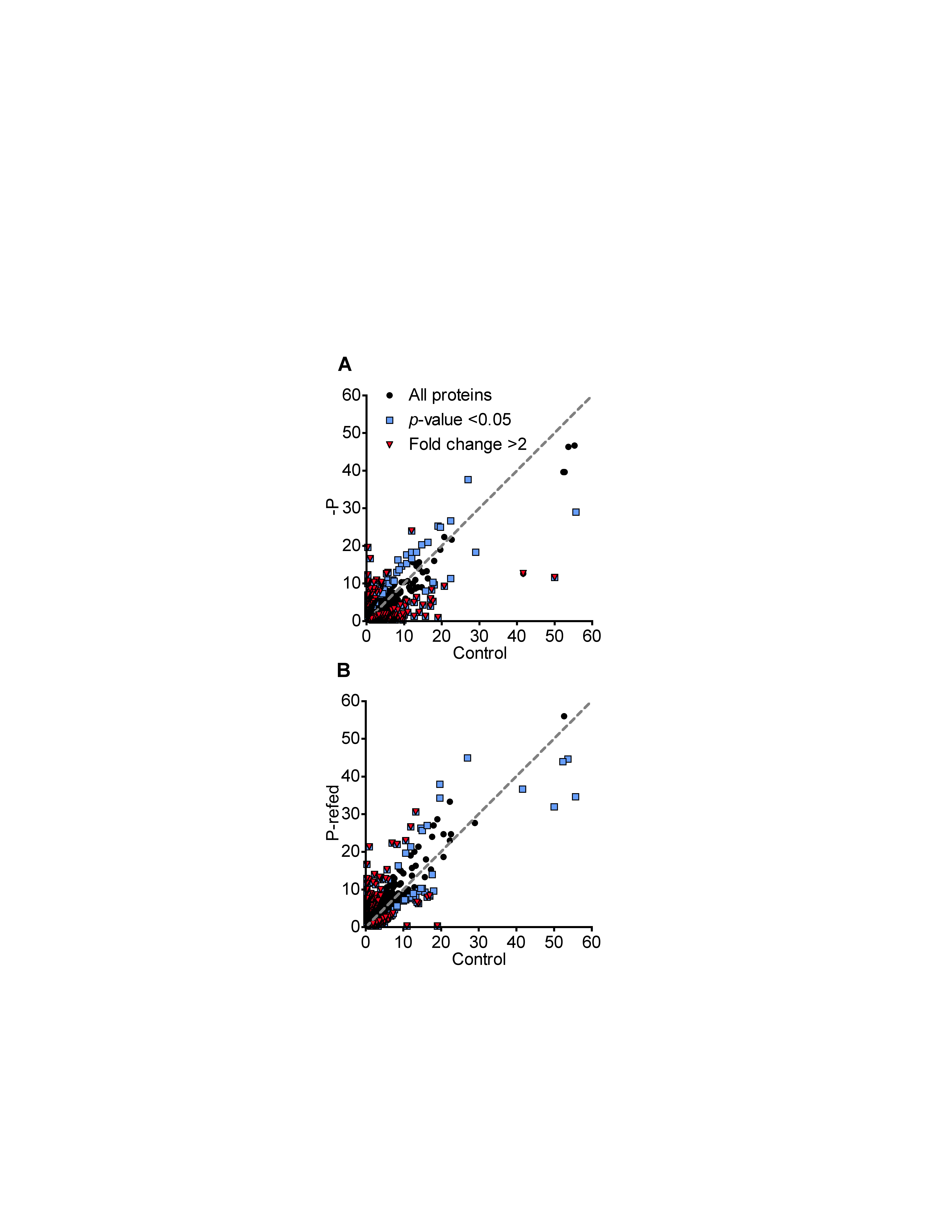

Supplement: Figure S1 — Protein abundances in each treatment. Scatter plot with the abundance of each protein in the (A) −P and control conditions and (B) P-refed and control conditions. Blue squares indicate proteins that are significantly different (p-value<0.05) between the conditions based upon the Fisher exact test. Red triangles specify proteins that are greater than 2-fold different between conditions. The gray dashed line indicates equal abundances between the conditions. (TIFF) [file pone.0028949.s001.tif]

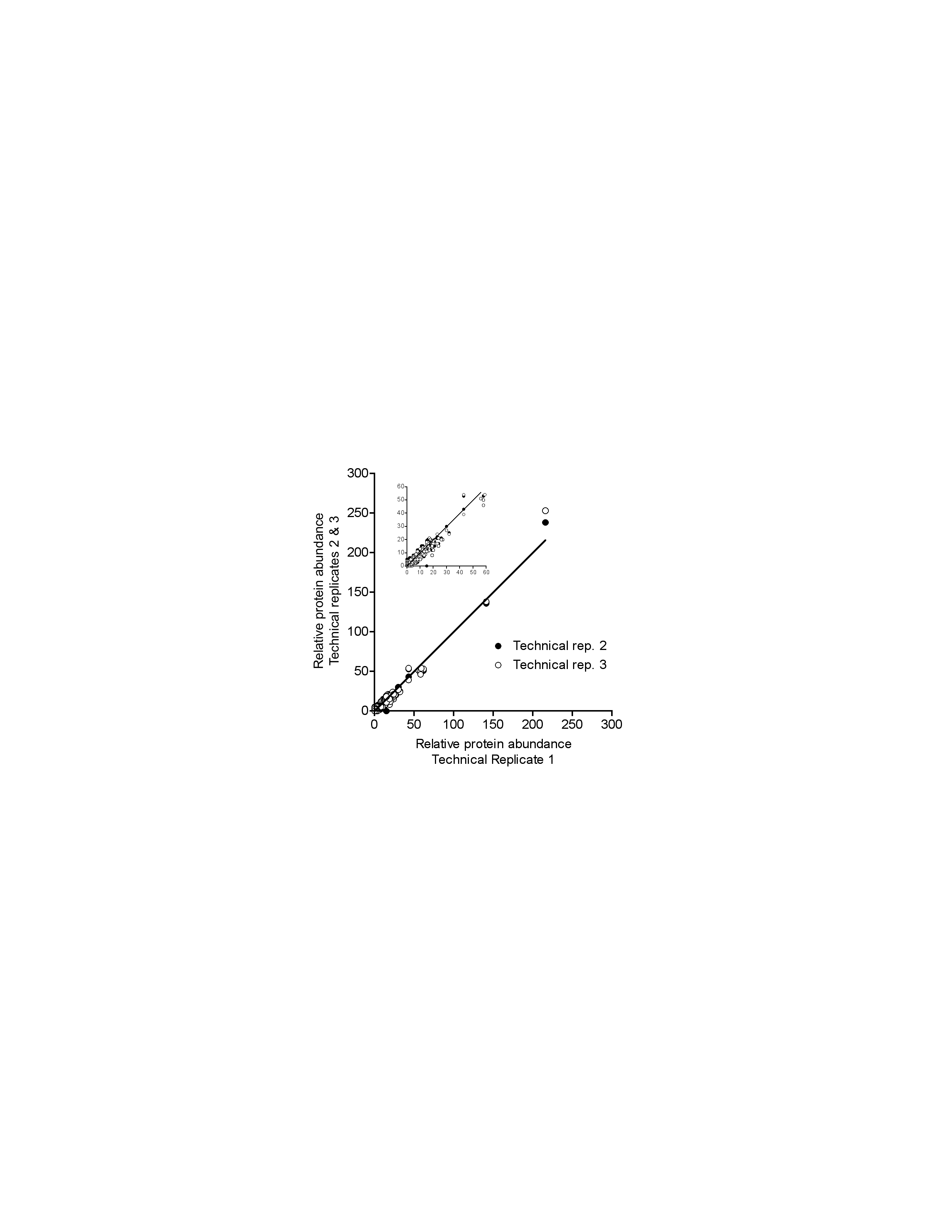

Supplement: Figure S3 — Spectral counting accuracy. Scatter plot demonstrating the precision of the method for spectral counting. Technical replicates of spectral count data from control conditions are plotted against each other. A 1∶1 line is shown for comparison. (TIFF) [file pone.0028949.s003.tif]
